# Regional AT-8 reactive tau species correlate with intracellular Aβ levels in cases of low AD neuropathologic change

**DOI:** 10.1007/s00401-024-02691-4

**Published:** 2024-02-14

**Authors:** Nauman Malik, Mohi-Uddin Miah, Alessandro Galgani, Kirsty McAleese, Lauren Walker, Fiona E. LeBeau, Johannes Attems, Tiago F. Outeiro, Alan Thomas, David J. Koss

**Affiliations:** 1 Translational and Clinical Research Institute, Faculty of Medical Sciences, Newcastle University, Newcastle Upon Tyne, UK; 2 Biosciences, Faculty of Medical Sciences, Newcastle University, Newcastle Upon Tyne, UK; 3 Department of Experimental Neurodegeneration, Center for Biostructural Imaging of Neurodegeneration, University Medical Center Goettingen, Göttingen, Germany; 4 Max Planck Institute for Multidisciplinary Sciences, Göttingen, Germany; 5 Division of Cellular and Systems Medicine, School of Medicine, University of Dundee, Dundee, Scotland, UK

**Keywords:** Alzheimer’s disease, Aβ, Intracellular Aβ, Tau pathology

## Abstract

The amyloid cascade hypothesis states that Aβ aggregates induce pathological changes in tau, leading to neurofibrillary tangles (NFTs) and cell death. A caveat with this hypothesis is the spatio-temporal divide between plaques and NFTs. This has been addressed by the inclusion of soluble Aβ and tau species in the revised amyloid cascade hypothesis. Nevertheless, despite the potential for non-plaque Aβ to contribute to tau pathology, few studies have examined relative correlative strengths between total Aβ, plaque Aβ and intracellular Aβ with tau pathology within a single tissue cohort. Employing frozen and fixed frontal cortex grey and white matter tissue from non-AD controls (Con; *n* = 39) and Alzheimer’s disease (AD) cases (*n* = 21), biochemical and immunohistochemical (IHC) measures of Aβ and AT-8 phosphorylated tau were assessed. Biochemical native-state dot blots from crude tissue lysates demonstrated robust correlations between total Aβ and AT-8 tau, when considered as a combined cohort (Con and AD) and when as Con and AD cases, separately. In contrast, no associations between Aβ plaques and AT-8 were reported when using IHC measurements in either Con or AD cases. However, when intracellular Aβ was measured via the Aβ specific antibody MOAB-2, a correlative relationship with AT-8 tau was reported in non-AD controls but not in AD cases. Collectively the data suggests that accumulating intracellular Aβ may influence AT-8 pathology, early in AD-related neuropathological change. Despite the lower levels of phospho-tau and Aβ in controls, the robust correlative relationships observed suggest a physiological association of Aβ production and tau phosphorylation, which may be modified during disease. This study is supportive of a revised amyloid cascade hypothesis and demonstrates regional associative relationships between tau pathology and intracellular Aβ, but not extracellular Aβ plaques.

**Supplementary Information:**

The online version contains supplementary material available at 10.1007/s00401-024-02691-4.

## Introduction

The original amyloid cascade hypothesis stated that the extracellular deposition of insoluble beta-amyloid (Aβ) plaques drives intracellular tau phosphorylation, the formation of neurofibrillary tangles (NFTs), and the subsequent neurodegeneration which underlies the pathology of Alzheimer’s disease (AD) [26]. Owing to the lack of correlation between plaque burden and cognitive impairment, as well as a growing understanding of the toxicity of fibrillar and pre-fibrillar intermediate species of Aβ and tau, the hypothesis has been revised to include roles for Aβ oligomers and tau oligomers [27]. Whilst the recent outcomes of plaque clearing and Aβ oligomer-targeted immunotherapies [19, 47, 67] support this revised amyloid cascade hypothesis, several inconsistencies relating to the interaction of Aβ and tau remain.

Foremost, is the spatio-temporal disconnect between the emergence and progression of Aβ plaque and tau NFT pathology. Based upon the post-mortem neuropathological Thal phases of Aβ deposition and positron emission tomography (PET) imaging studies, Aβ plaques were found to originate within the neocortex, specifically within the orbito-frontal and medial parietal cortices, before spreading to the hippocampus, the brain stem and cerebellum [46, 63]. In contrast, as reflected by Braak NFT staging, tau pathology initially occurs in the entorhinal cortex and hippocampus and subsequently spreads to the lateral temporal and parietal cortices and finally to the frontal and occipital cortices [10, 13].

Cross-sectional population studies further highlight the independent nature of the two hallmark pathologies, reporting that tau pathology consistent with Braak stages I–II occurs more readily with age than that of plaque deposition [62]. Consequently, Aβ deposition is not a prerequisite for NFT formation in ageing or in case of primary tauopathies [76] and primary age-related tauopathies (PART) [18]. Moreover, the demonstration of prion-like spreading via tau seed templating and pathology propagation provides a mechanistic process by which the presence of tau pathology may occur independent from the influence of Aβ plaques [21]. Such tau seed propagation of pathology may contribute to the progression of tau pathology in many tauopathies, including AD, although it should be noted that at present evidence for the occurrence of this self-propagation is based on supra-physiological levels of tau seeds [50].

Taken together, the direct causation of NFTs, purely as a consequence of Aβ plaque burden is difficult to ratify, with the differential emergence in time and space of the neuropathological hallmarks, as well as the independent occurrence of tau aggregations in other neurodegenerative conditions.

However, it remains likely that plaque deposition, or rather the process of amyloid plaque formation, influences the generation of tau pathology. This is perhaps most strongly supported by numerous biochemical studies of human brain tissue, which report robust correlations with pathological Aβ and tau species [30, 39, 41, 54]. Despite the close relationship between tau and Aβ levels in various biochemical assays, immunohistochemical (IHC) approaches frequently fail to detect such correlations. The disconnect between biochemical and IHC analysis clearly highlights differences in the pathological species measured within the different methodological approaches.

In line with the revised amyloid cascade hypothesis [27], a range of experimental models demonstrate that soluble pathological species of both tau and Aβ exert toxic influence within the brain, evident in rodent in vivo injection models [9, 38, 44, 55], familial AD (FAD) [5, 14, 65] and tauopathy [6, 37, 40] mouse models and cell culture approaches [15, 34]. Whilst APP-centric FAD mouse models do not develop NFTs, there is clear evidence of increased tau phosphorylation within these models [51, 58, 65]. Moreover in multi-genic mice, in which a human mutant tau gene is included, APP-centric mutations can accelerate and enhance NFT pathology [28, 60]. Nevertheless, evidence for the induction of tau pathology following Aβ intracerebral delivery in vivo is sparse in non-transgenic animals [3, 4] and even in tau transgenic mice [8].

Whilst there are several possible explanations for the failure of exogenous Aβ to drive tau pathology in vivo, one possible contributing factor is that the sole delivery of Aβ to the extracellular space may not be sufficient to drive tau pathology. Indeed, a growing body of evidence suggests that intracellular Aβ accumulation may also play a role in the pathobiology of AD [1], influencing cellular dysfunction [56, 59, 66] and tau phosphorylation [65]. Notably, the familial AD Osaka E639Δ APP mutation, produces non-fibril E22Δ Aβ and gives rise to an accumulation of intracellular Aβ oligomers in the absence of plaques [64]. In AD patients or mouse models carrying the Osaka mutation, pronounced cognitive impairments, cellular stress, synaptic spine loss, and, critically, pathological tau phosphorylation and conformational changes are observed [33, 65].

Despite the potential for non-plaque Aβ to contribute to the production of tau pathology, few studies have sought to examine both biochemical and immunohistochemical quantification of Aβ and tau pathology within the same human post-mortem cases, thus, allowing for a direct comparison between the correlative strength of total Aβ, plaque Aβ and intracellular Aβ with tau pathology. This current study aimed to quantify such parameters in control cases with low AD-related neuropathological change as well as in AD subjects. Such measures were used to establish potential correlative relationships between various pathological Aβ sources and regional tau pathology.

## Methods

### Human post-mortem brain tissue

A study cohort of post-mortem human brains from clinico-pathologically classified AD (*n* = 21) and non-neurodegenerative control cases (Con, *n* = 39) was obtained from the Newcastle Brain Tissue Resource (NBTR). AD subjects had been clinically assessed during life, prior to brain tissue donation, and diagnosed with dementia due to AD. Control cases similarly had been assessed during life and, at the time of death, did not have dementia. The final clinico-pathological diagnoses were established by combining clinical neuropathological data reviewed at regular meetings involving JA and AT. Neuropathological diagnoses were based on assessment of brain tissue according to the National Institute of Ageing-Alzheimer’s Association (NIA-AA) criteria [52], including Braak NFT staging [12], Thal phases [63], and Consortium to Establish a Registry for Alzheimer’s Disease (CERAD) scoring [31], as well as Braak LB stages [11] and Newcastle/McKeith criteria [48, 49] (Table 1; Supplemental Table 1, online resource, for full details).

**Table 1:** Post-mortem human tissue cases and use

Disease	*N*	Sex (% male)	Age (years)	PMI (hrs)	NFT Braak stage	Thal phase	CERAD	NIA-AA	LB Braak stage	McKeith Criteria
Immunoblots (AT-8 and MOAB-2)										
Con	35	57.1%	47–97 80.5 ± 2.2	16–95 45.8 ± 3.7	0-IV 17.1%-0 17.1%-I 25.7%-II 25.7%-III 14.3%-VI	0–4 20%-0 22.9%-1 25.7%-2 22.9%-3 8.6%-4	0–2 80%-0 5.7%-1 14.3%-2	0–2 20%-0 65.7%-1 14.3%-2	0–3 91.4%-0 2.9%-1 5.7%-3	91.4%-No LB 8.6%-Brainstem
AD	17	35.3%	74–96 85.5 ± 1.5	5–90 57.2 ± 5.7	V-VI 29.4%-V 70.6%-VI	4–5 15.4%-4 84.6%-5	3 100%-3	3 100%-3	0–3 82.3%-0 11.8%-2 5.9%-3	76.5%-No LB 17.6%-Brainstem 5.9%-Limbic
Immunohistochemistry (AT-8 and 48G plaques)										
Con	36	56.2%	55–97 81.5 ± 2.2	16–95 47.8 ± 3.9	0-IV 16.7%-0 19.4%-I 25%-II 25%-III 13.9%-VI	0–4 16.7%-0 25%-1 30.6%-2 19.4%-3 8.3%-4	0–2 77.8%-0 8.3%-1 13.9%-2	0–2 16.7%-0 69.4%-1 13.9%-2	0–3 88.1%-0 5.8%-1 6.1%-3	88.7%-No LB 8.3%-Brainstem 2.8%-Limbic
AD	20	30%	70–93 85 ± 1.3	5–90 54.4 ± 4.9	V-VI 25%-V 75%-VI	4–5 13.9%-4 86.1%-5	3 100%-3	3 100%-3	0–3 85%-0 10%-2 5%-3	80%-No LB 15%-Brainstem 5%-Limbic
Immunohistochemistry (AT-8 and MOAB-2 intracellular Aβ)										
Con	13	30.8%	70–97 84.7 ± 2.4	16–95 47 ± 7.6	0-IV 7.7%-0 15.4%-I 38.4%-II 38.4%-III	0–2 30.8%-0 38.4%-1 30.8%-2	0–1 92.3%-0 7.7%-1	0–1 38.5%-0 61.5%-1	0–3 84.6%-0 15.4%-3	79.6%-No LB 15.4%-Brainstem 7.7%-Limbic
AD	9	22.2%	78–93 87 ± 1.7	29–90 61.1 ± 7	V-VI 11.1%-V 88.9%-VI	5 100%-5	3 100%-3	3 100%-3	0–3 77.8%-0 22.2%-2	77.8%-No LB 22.2%-Brainstem

Human cases use for immunoblots and immunohistochemistry for plaques and AT-8 as well as intracellular Aβ and AT-8 are listed. Cases are separated by disease classification according to non-diseased controls (Con) and Alzheimer’s disease (AD). Case numbers (*n*), sex, age, post-mortem interval (PMI), neurofibrillary tangle (NFT) Braak stage, Thal phase, Consortium to Establish a Registry for Alzheimer’s Disease (CERAD), the National Institute of Ageing-Alzheimer’s Association (NIA-AA) criteria, Lewy body (LB) Braak stage and McKeith criteria are provided. For age and PMI both range and mean ± SEM are provided. For numerical scores of pathology, range and percentage composition are given. For CERAD scores, negative (neg) A and B reported. For NIA-AA, not, low and intermediate (inter) risk for Alzheimer’s disease. For McKeith criteria, only percentage composition is given, where cases free of LBs (no LB), brainstem, limbic and neocortex (Neo) predominate are indicated

For histology and tissue micro-array (TMA; see below), tissue sections were prepared from the right hemisphere of the brain and fixed for 4–6 weeks in 4% paraformaldehyde. Corresponding frozen frontal grey (GM) and white matter (WM) tissue (Brodmann’s area (BA) 9) was obtained from the left hemisphere, dissected in a coronal plane and snap frozen between copper plates at −120 °C prior to being stored at −80 °C. Due to limitations in tissue availability, it was not possible to obtain both fixed and frozen tissue for all cases (see Table 1; Supplemental Table 1, online resource, for full details). Comparative analysis of age and post-mortem interval (PMI) between disease groups determined there was no significant difference in either measure (*p* > 0.05).

### Tissue lysis

 ~ 250 mg of frozen frontal tissue was electronically homogenized 1:10 (W/V) in 0.2 M tetraethyl ammonium bicarbonate (TEAB, pH 7.2, Sigma) with 1% SDS, containing protease (1 per 10 ml, Complete, Roche) and phosphatase inhibitors (1 per 10mls, PhosSTOP, Sigma) using an Ultra-turrax T10 homogenizer (5 mm diameter probe; 30,000 rpm) for 15 s. Lysates were aliquoted and stored at −80 °C, prior to use.

### Immunoblot quantification of AD markers

Dot blots were conducted for total Aβ and AT-8 phospho-tau in both GM and WM samples. The protein concentrations of GM and WM crude lysate were adjusted to 0.5 µg/µl as per Bradford assay and dotted directly to a nitrocellulose membrane at 10 μl (5 µg/dot) and left to dry for 20 min before further processing. The membranes were briefly washed in Tris-buffered saline (TBS; in mM; 50 Trizma base, 150 NaCl, pH = 7.6) prior to being blocked in 5% milk powder containing Tris-buffered saline with 0.1% Tween 20 (TBST) at room temperature for 1 h. After blocking, blots were rinsed in TBS washing buffer 3 times for 5 min each. Membranes where subsequently placed in primary antibody solution (TBST, 5% bovine serum albumin and 0.05% sodium-Azide) containing either MOAB-2 (1:1000, Cat# M-1586-100, Biosensis) for the detection of Aβ or AT-8 (1:1000, Cat# AB_223647, Thermofisher) for phospho-tau and incubated overnight at 4 °C. The membranes were then washed in TBST before being incubated for 1 h at room temperature in horse radish peroxidase conjugated goat anti-mouse secondary IgG antibody (TBST + 5% milk powder + 1:5000 dilution) prior to repeated washing before being developed. Immunoreactivity was visualized via enhanced chemiluminescence (ECL; 1.25 mM luminol, 25 μl of 3% H202 and 50 μl coumaric acid was incubated for 1 min). The signal was captured by using a digital western blot camera. The images were saved as 8-bit for illustration and 16-bit for quantification. Total protein loading was determined via Ponceau S general protein stain (0.1% Ponceau S (w/v) and 5.0% acetic acid (w/v) in ddH_2_O) and resulting loading staining captured.

### Immunoblot quantification

Immunoreactivity and Ponceau S-stained blots were quantified from 16-bit digitized images based on area under the curve measurements as computed by ImageJ (Ver 1.53e, NIH, USA). Normalization of immunoblot intensity values were then performed using total protein adjusted values. The 52 samples of human frontal cortex GM and WM were processed in 4 separate batches and each batch normalized to the mean value of control cases (each blot containing > 3 Braak stage 0–IV control cases) prior to pooling values between blots.

### Immunohistochemical quantification of Aβ plaques and phospho-tau (AT-8)

Regional quantification of the Aβ plaque and AT-8 phospho-tau load within the frontal cortex (B A9) was preformed via TMA slides, as described previously [68]. Sections (6 µm thick) were cut from paraffin-embedded TMA blocks tissue blocks comprising cylindrical tissue cores taken from multiple brain region-specific blocks and mounted on glass slides. Slides containing 3 mm diameter samples of BA9 frontal cortex were baked at 60 °C for 1 h prior to being dewaxed in xylene, rehydrated in descending concentrations of ethanol (5 min immersion) and washed in TBS. Slides intended for phospho-tau staining were treated with microwave-assisted antigen retrieval (800 W, 10 min) in citrate buffer (10 mM citric acid, 0.05% Tween 20, pH 6) and those intended for Aβ plaque staining were submerged in 90% Formic acid for 1 h at RT, before endogenous peroxidases were quenched in H_2_O_2_ (3%, 20 min submersion). Following consecutive washes in TBS and TBST, slides were incubated with either mouse 4G8 (1:16000, Cat# SIG-39200, Covance) or anti-AT-8 (1:4000) in TBS for 1 h and immunoreactivity visualized via the MENAPATH HRP polymer detection kit (Menarini diagnostics, Wokingham, UK) and 3,3′-diaminobenzidine (DAB) chromogen with appropriate TBS and TBST washes performed between steps. Slides were co-stained with haematoxylin prior to being dehydrated in ethanol, cleared in xylene and mounted in dibutylphthalate polystyrene xylene (DPX).

Stained BA9 frontal cortex samples were imaged at 100× magnification with a semi-automated microscope (Nikon Eclipse 90i microscope, DsFi1 camera and NIS elements software V 3.0, Nikon). For each case, multiple images were captured to form a 3 × 3 image grid with 15% overlap in adjacent images, such that an area of 1.7 mm was sampled from each case.

Following visual quality control inspection and the application of regions of interest (ROI) to exclude areas of tissue folds and tears, a consistent restriction threshold for 4G8 (R50-180, G20-168, and B8-139) and AT-8 (R25-170, G27-156, B11-126) was applied producing a binary signal image from which the percentage area of immunoreactivity could be acquired. For the quantification of Aβ plaques, 4G8 images were further processed by means of size exclusion, restricting object detection to > 100µm^2^, thus avoiding inclusion of intracellular APP and Aβ.

### Immunofluorescent histochemical analysis of intracellular Aβ and phospho-tau (AT-8)

Paraffin-embedded tissue blocks of the frontal cortex BA9 were used to prepare sections (6 µm thick) for the purpose of multiplex intracellular Aβ and phospho-tau fluorescent staining. Slide-mounted frontal cortex sections were baked at 60 °C for 1 h, dewaxed and rehydrated and subjected to antigen retrieval in citrate buffer and formic acid treatment (as above). Slides were then blocked in TBST containing 10% normal goat serum for 1 h at RT and incubated in mouse IgG2b anti-MOAB-2 and mouse IgG1 anti-AT-8 (1:500, for both) overnight at 4 °C, prior to incubation in secondary antibodies (goat anti-mouse IgG1 Alexa 488 and goat anti-mouse IgG2b Alexa 594, 1:1000 for both, Invitrogen). Endogenous tissue fluorescence was quenched via post-staining treatment with Sudan black (0.01%, 70% ethanol, 5 min submersion) before slides were coverslipped with DAPI-containing Prolong Diamond Mounting media (Fisher Scientific). In a subset of slides, the limited colocalization of MOAB-2 labelled Aβ and APP was established, staining sections with mouse-IgG2b anti-MOAB-2 and rabbit-anti-APP (1:500, Cat# ab15272, Abcam) and appropriate secondary antibodies. Fluorescence antibody-labelled sections were imaged via a wide-field fluorescence microscope system (Nikon Eclipse 90i microscope, DsQi1Mc camera and NIS elements software V 3.0, Nikon).

One section per case was examined at 400× magnification with three images per grey matter and white matter regions selected at random. As these images were used for quantification of intracellular Aβ, excluding Aβ-plaques, any region selected which contained multiple plaques was excluded and another region selected. ROI were manually applied to each image and folds and tears and plaques were excluded, before images were converted to greyscale and a consistent threshold applied to generate a binary image from which percentage area of immunoreactivity was determined. The mean percentage area of immunoreactivity was calculated per grey and white matter area per case.

### Data analysis

Data were subjected to Shapiro–Wilk normality tests for normal distributions, prior to statistical comparison between control and AD cases using a non-parametric Mann–Whitney *U* test (GraphPad Prism Ver. 5). In SPSS, two-tailed Spearman’s correlation was used for correlation analysis. Given the association of increasing Braak stage with age, all correlations with Braak staging were performed with partial correlations controlling from age. A series of one-tailed *t* test was performed to identify the initial stage at which measures were significantly elevated from Braak 0 pathological controls. Identification of outliers was conducted via Grubb’s test, with any outliers removed clearly noted in the results. For all analysis, *p* < 0.05 was considered as statistically significant, with increasing statistical reliability for *p* < 0.01, *p* < 0.001 and *p* < 0.0001.

## Results

### Biochemical analysis of total Aβ and phospho-tau pathology

To limit the potential confounding influence of age-related Aβ-independent tau pathology as seen in PART [18], Brodmann’s area 9 of the superior frontal cortex, a region which does not develop NFTs until late-stage AD-related disease progression (Braak NFT V-VI) was selected for investigation. Crude tissue lysates were employed to ensure that no specific Aβ/tau pools (soluble/insoluble) were lost from the samples. Equally the use of dot blots, whilst preventing the quantification of specific oligomeric species, avoided the confounding effects off SDS exposure, which has been shown to modify Aβ oligomerisation state [69]. Similarly, the use of dot blots also mitigated sample modification by heat exposure, which has previously been shown to result in a loss of meaningful MOAB-2 based immunoreactivity from tissue lysates [39].

Accordingly, dot-blot quantification of crude tissue lysates of grey matter of this region as well as the associated white matter of AD subjects (*n* = 17; Braak stage V-VI) and non-AD controls (*n* = 35; Braak stage 0-IV) were probed for total Aβ (intracellular and extracellular species) via the non-APP cross-reactive MOAB-2 antibody [74] and for tau pathology using the phospho-tau-specific antibody AT-8.

**Fig. 1:**
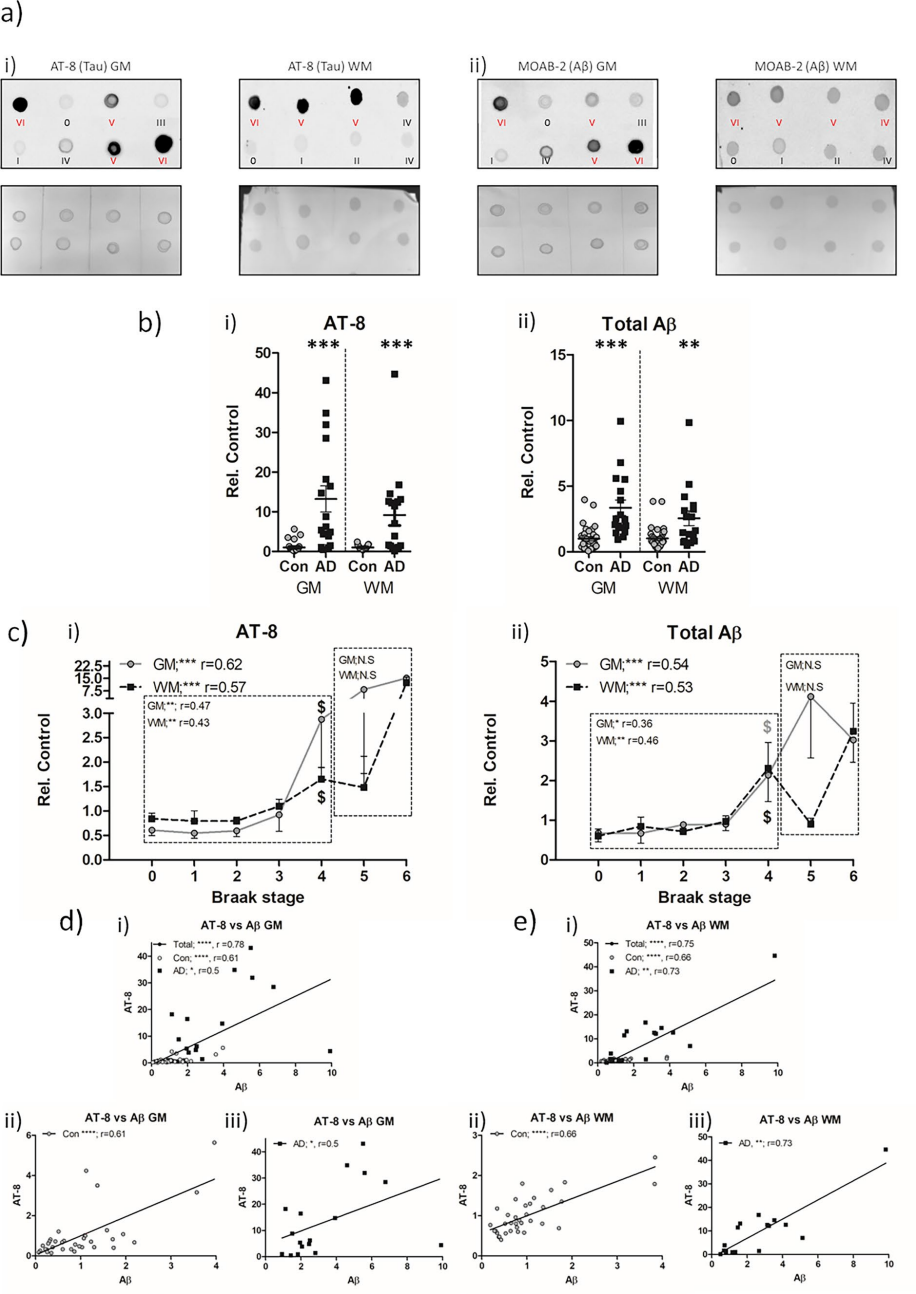
Biochemical quantification of AT-8 phospho-tau and total Aβ in the frontal cortex of non-AD and AD cases. **a** Example dot blots of AT-8 (*i*) and MOAB-2 (Aβ; *ii*) immunoreactivity and associated Ponceau total protein stain, produced from crude tissue lysates of frontal grey (GM) and white matter (WM) in control (Con; black lettering) and Alzheimer’s disease (AD; red lettering) cases. Braak NFT stage of each sample is shown. **b** Comparison of mean AT-8 (*i*) and Aβ (*ii*) immunoreactivity between Con (*n* = 35) and AD (*n* = 17) cases in the GM and WM. **c** Association of AT-8 (*i*) and Aβ (*ii*) immunoreactivity with Braak NFT stages across the cohort in GM and WM. Correlative analysis (Spearman’s *r*) is shown for when analysis as a single group or when separated into Con and AD groups. Combined (*i*), Con (*ii*) and AD (*iii*) linear correlations between AT-8 and Aβ in the GM (**d**) and WM (**e**). Immunoreactivity shown as relative to control (Rel. Control). * = *p* < 0.05, ** = *p* < 0.01, *** = *p* < 0.001 and **** = *p* < 0.0001. $ denotes initial Braak NFT stage at which immunoreactivity is significantly elevated from Braak 0 controls

When considered purely based on the neuropathological diagnosis of either non-AD controls versus AD, levels of AT-8 phospho-tau were elevated in AD cases, both in GM (13.24 ± 3.3 fold cf. non-AD, *p* < 0.001, Fig. 1a.i + b.i) and WM (9 ± 2.6 fold cf. non-AD, *p* < 0.001, Fig. 1a.i + b.i). Despite a numerically higher mean within the GM compared to the WM, there were no statistically significant differences between the magnitude of increase between GM and WM in AD cases (*p* > 0.05). Similarly, when Aβ levels were examined based on the neuropathological cohort stratification of non-AD controls and AD, elevations were apparent within the GM (3.35 ± 0.59 fold c.f. non-AD, *p* < 0.01, Fig. 1a.ii + b.ii) and WM (2.56 ± 0.56 fold c.f. non-AD, *p* < 0.05, Fig. 1a.ii + b.ii) of the AD cases. Again, no difference between the magnitude of increase within AD cases relative to control cases was observed between GM and WM (*p* > 0.05).

Such an outcome from the analysis of phospho-tau and Aβ between AD and non-AD control cases is not surprising, but serves to validate the use of dot blots to measure biochemical changes in phospho-tau and Aβ.

To further place the observed changes of tau and Aβ within the context of disease progression, the entire cohort (non-AD and AD cases) was subdivided based on the respective Braak stages (0-VI) and crude lysate measures of AT-8 phospho-tau and Aβ correlated with disease progression (Fig. 1c.i + ii). Phospho-tau AT-8 immunoreactivity correlated with increasing Braak stages when considered across the entire cohort and adjusted for age (Fig. 1c.i) within the GM (*r* = 0.62, *p* < 0.001) as well as in the WM (*r* = 0.57, *p* < 0.001). Interestingly, when probed for the stage at which phospho-tau levels were significantly elevated from that of “pathologically free” Braak stage 0 cases, Braak stage IV, the stage prior to the gross involvement of the frontal cortex with NFT pathology, was indicated (*p* < 0.05, for both GM and WM). Furthermore, when split according to neuropathological diagnosis, and controlled for age, a significant correlation was observed between AT-8 phospho-tau and Braak stage in the non-AD control group (Braak 0-IV) in GM (Fig. 1c.i, *r* = 0.47, *p* < 0.01) and WM (Fig. 1c.i, *r* = 0.43, *p* < 0.05), but not in AD cases (Braak V-VI, Fig. 1c.i, *p* > 0.05). Whilst the failure to find a correlation of AT-8 phospho-tau in the AD cases may be due to a ceiling effect of pathology or indeed the correlation of variables across only two Braak stages, it is nevertheless striking that in non-AD control cases, AT-8 phospho-tau increases within the frontal cortex in line with the inter-regional spatial progression of NFTs.

Following a similar line of investigation for the accumulation of Aβ in relation to disease progression, Aβ levels were correlated with individual Braak NFT stages. Across the entire cohort of control and AD cases (Braak stage 0-VI), robust correlations were reported for both the GM (*r* = 0.54, *p* < 0.001, Fig. 1c.ii) and WM (*r* = 0.53, *p* < 0.001, Fig. 1d), when controlling for age. In line with observations of AT-8 phospho-tau, individual comparisons with Braak 0 cases reported an initial significant elevation from the “pathologically free” baseline at Braak IV, in GM and WM samples (*p* < 0.05). When divided into non-AD control (Braak stage 0–IV) and AD (Braak stage V–VI) groups, increasing total Aβ correlated with progressive Braak stages in the non-AD group in both the GM and WM (*r* = 0.36, *p* < 0.05 and *r* = 0.46, *p* < 0.01 in GM and WM, respectively, Fig. 1c.ii), but not in the AD group (*p* > 0.05).

Collectively, the data demonstrates that in the frontal cortex, intra-regional tau and total Aβ pathology progress in accordance with global AD-related tau pathology, which is surprisingly most apparent in non-AD controls compared to AD cases (see Table 2 for summary).

**Table 2:** Summary correlation table of total plaque and intracellular Aβ with AT-8 phospho-tau

	Braak	Age	PMD	AT8-GM	AT8-WM	Total-Aβ-GM	Total-Aβ-WM
**Biochemical dot-blot total AT-8 tau and Aβ**							
*Total cohort*							
AT8-GM	*r* = 0.62***	N.S.	N.S.		*r* = 0.71**	*r* = 0.78****	*r* = 0.56**
AT8-WM	*r* = 0.57***	N.S.	N.S.	*r* = 0.71**		*r* = 0.54**	*r* = 0.75**
Total-Aβ-GM	*r* = 0.54***	N.S.	N.S.	*r* = 0.78**	*r* = 0.54**		*r* = 0.61**
Total-Aβ-WM	*r* = 0.53***	N.S.	N.S.	*r* = 0.56**	*r* = 0.75**	*r* = 0.61**	
*Controls*							
AT8-GM	*r* = 0.47**	N.S.	N.S.		*r* = 0.48**	*r* = 0.61****	N.S.
AT8-WM	*r* = 0.43**	N.S.	N.S.	*r* = 0.47**		*r* = 0.37*	*r* = 0.66**
Total-Aβ-GM	*r* = 0.36*	N.S.	N.S.	*r* = 0.61**	*r* = 0.37*		*r* = 0.59**
Total-Aβ-WM	*r* = 0.46**	N.S.	N.S.	N.S.	*r* = 0.66**	*r* = 0.59**	
*Alzheimer’s disease*							
AT8-GM	N.S.	N.S.	N.S.		*r* = 0.67**	*r* = 0.5*	N.S.
AT8-WM	N.S.	N.S.	N.S.	*r* = 0.67**		N.S.	*r* = 0.73**
Total-Aβ-GM	N.S.	N.S.	N.S.	*r* = 0.5*	N.S.		**r = 0.6
Total-Aβ-WM	N.S.	N.S.	N.S.	N.S.	*r* = 0.73**	**r = 0.6	
**IHC—plaques**							
*Total cohort*							
AT8	*r* = 0.49****	N.S.	N.S.		*r* = 0.68**		
Plaques	*r* = 0.65**	N.S.	N.S.	*r* = 0.68**			
*Controls*							
AT8	N.S.	*r* = 0.36*	N.S.		N.S.		
Plaques	N.S.	N.S.	N.S.	N.S.			
*Alzheimer’s disease*							
AT8	N.S.	N.S.	N.S.		N.S.		
Plaques	N.S.	N.S.	N.S.	N.S.			
**IHC—AT8 tau and intracellular Aβ**							
*Total cohort*							
AT8-GM	*r* = 0.60**	N.S.	N.S.		*r* = 0.77**	*r* = 0.72**	*r* = 0.44*
AT8-WM	*r* = 0.53*	N.S.	N.S.	*r* = 0.77**		*r* = 0.73**	**r = 75
Intracell-Aβ-GM	*r* = 0.44*	N.S.	N.S.	*r* = 0.72**	*r* = 0.73**		*r* = 0.74**
Intracell-Aβ-WM	N.S.*	N.S.	N.S.	*r* = 0.46*	*r* = 0.75**	*r* = 0.74**	
*Controls*							
AT8-GM	N.S.	N.S.	N.S.		*r* = 0.69**	*r* = 0.83**	N.S.
AT8-WM	N.S.	N.S.	N.S.	*r* = 0.69**		*r* = 0.68**	*r* = 0.72**
Intracell-Aβ-GM	N.S.	N.S.	N.S.	*r* = 0.83**	*r* = 0.68**		*r* = 0.56*
Intracell-Aβ-WM	N.S.	N.S.	N.S.	N.S.	*r* = 0.72**	*r* = 0.56*	
*Alzheimer’s disease*							
AT8-GM	N.S.	N.S.	N.S.		N.S.	N.S.	N.S.
AT8-WM	N.S.	N.S.	N.S.	N.S.		N.S.	N.S.
Intracell-Aβ-GM	N.S.	N.S.	N.S.	N.S.	N.S.		N.S.
Intracell-Aβ-WM	N.S.	N.S.	N.S.	N.S.	N.S.	N.S.	

Correlation matrix, reporting the significance and strength of Spearman’s (*r*) correlative relationships between biochemical and immunohistochemical (IHC) Aβ and phospho-tau (AT-8) measures in the frontal grey and white matter. Additional correlations with experimental measures and Braak stage, age and post-mortem delay (PMD) are reported. Data is presented for correlations preformed as the entire cohort as a whole and when spilt into controls and Alzheimer’s diseases cases only

^*^ = *p* < 0.05, ** = *p* < 0.01, *** = *p* < 0.001 and **** = *p* < 0.0001. N.S. = not significant

A critical element to this regional pathology is to determine whether biochemical measures of tau pathology and Aβ correlate on a case-by-case basis, as the disconnect between IHC tau and Aβ pathological hallmarks has long been a major caveat to the amyloid cascade hypothesis. Indeed, a robust correlation between biochemical measures of AT-8 phospho-tau and total Aβ measures was observed when considered as a single cohort (non-AD + AD cases, Braak stage 0-VI) in the GM (*r* = 0.78, *p* < 0.0001, Fig. 1d.i) and in the WM (*r* = 0.75, *p* < 0.0001, Fig. 1e.i). Remarkably, when examined separately within non-AD cases (Braak stage 0-IV) and AD cases (Braak V–VI), correlations between AT-8 phospho-tau and Aβ were apparent in both control (GM; *r* = 0.61, *p* < 0.0001 and WM; *r* = 0.66, *p* < 0.0001, Fig. 1d.ii + e.ii) as well as AD cases (*r* = 0.5, *p* < 0.05 and *r* = 0.73, *p* < 0.001 in GM and WM, Fig. 1 d.iii + e.iii). Additional correlations were observed between GM tau and Aβ measures and those obtained from the WM (see Table 2 for summary).

Thus, the data here is in support of regionally generated tau pathology, driven by regional Aβ accumulation, not only in AD cases, but also across a spectrum of control cases with varied AD-related neuropathic change.

### Histochemical quantifications of AT-8 phospho-tau and Aβ plaques

**Fig. 2:**
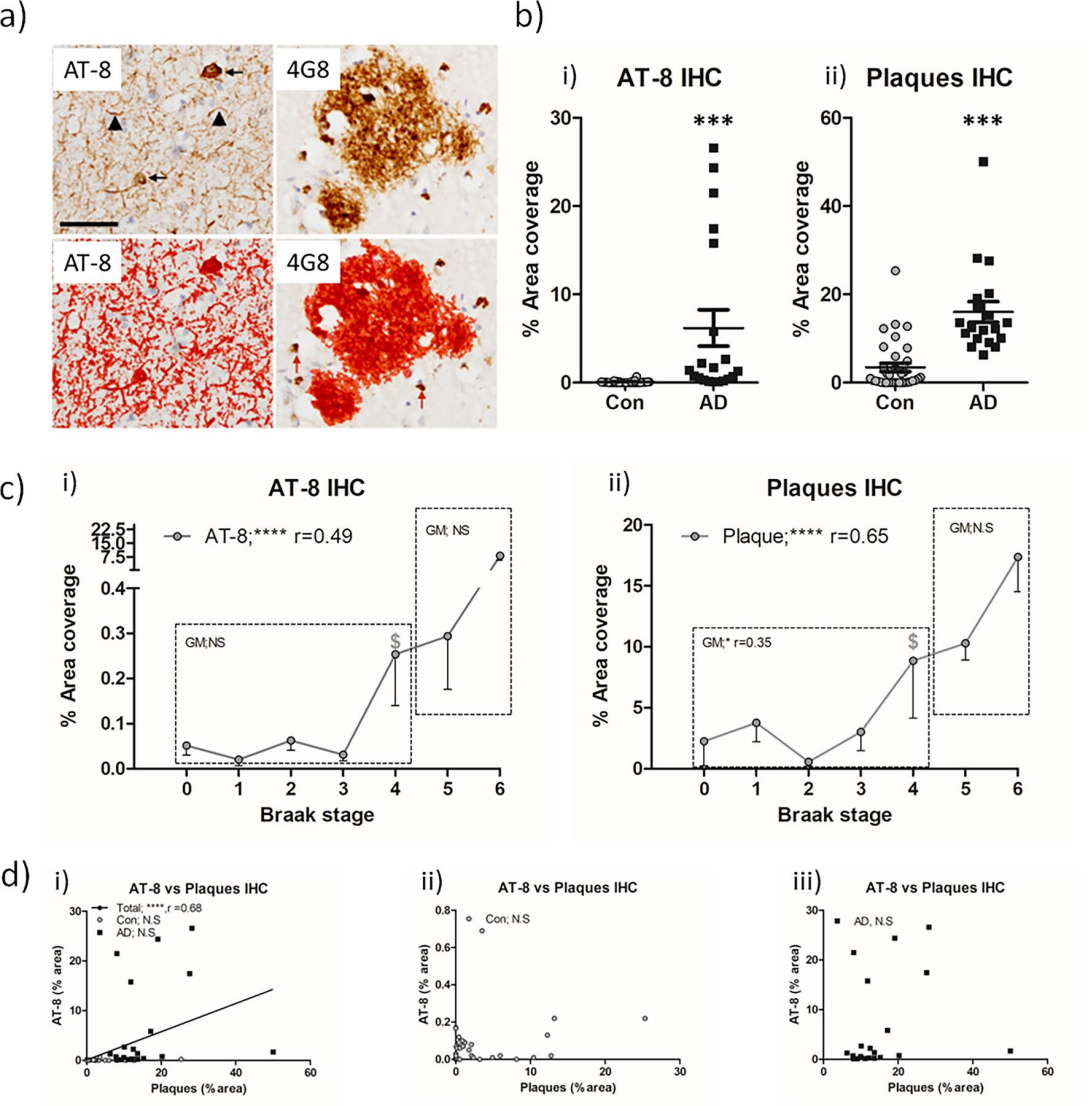
Immunohistochemical quantification of AT-8 tau and Aβ plaque burden in the frontal cortex of non-AD and AD cases. **a** Example micrographs of DAB based AT-8 and 4G8 (Aβ) immunoreactivity, area of quantification following threshold application shown in red. Note the size exclusion in this parameter of intracellular 4G8 labelling to negate potential APP cross-reactivity (arrows). **b** Quantification of % area coverage of AT-8 (*i*) and plaque (*ii*) immunoreactivity in control (Con, *n* = 36) and Alzheimer’s disease (AD, *n* = 20) cases. **c** Association of % area coverage of AT-8 (*i*) and plaques (*ii*) with Braak NFT stage with correlative analysis (Spearman’s *r*) shown. Combined (*i*), Con (*ii*) and AD (*iii*), linear correlations between AT-8 and plaques in the GM (D). N.S. = not significant, *** = *p* < 0.001 and **** = *p* < 0.0001. $ denotes initial Braak NFT stage at which immunoreactivity is significantly elevated from Braak 0 controls. Scale in a = 20 µm

To establish if the biochemically derived relationship of increased Aβ immunoreactivity, correlating with increased AT-8 immunoreactivity, was primarily driven by an association of Aβ plaques with AT-8 phospho-tau, semi-quantitative immunohistochemistry analysis was performed. TMA slides from all cases were stained with either anti-Aβ antibody 4G8 or AT-8 phospho-tau. For quantitation of Aβ plaques, all intracellular immunoreactivity as detected via the APP cross-reacting 4G8 antibody was excluded (Fig. 2a; see Supplemental Table 1 for details). Based on % area coverage, AT-8 immunoreactivity, a composite of NFTs and NTs showed a marked ~ 100-fold increase in % coverage in the frontal grey matter of AD cases compared to controls (7.6 ± 2.5% cf. 0.07 ± 0.02%, in AD and non-AD cases respectively, *p* < 0.001, Fig. 2b.i). Equally, quantification of the % area coverage of Aβ plaques between AD cases and non-AD controls, also, unsurprisingly reported a significant increase with the AD cases (14.26 ± 1.7% cf. 3.55 ± 1%, *p* < 0.001, Fig. 2b.ii).

When measures were considered in relation to progressive Braak NFT staging across the entire cohort (Braak stage 0-VI), AT-8 phospho-tau (*r* = 0.49, *p* < 0.0001, Fig. 2c.i) and Aβ plaques (*r* = 0.67, *p* < 0.0001, Fig. 2c.ii) strongly correlated with Braak stage, following a correction for age. Again, a significant elevation in AT-8 phospho-tau and Aβ plaque coverage from “pathologically free” Braak stage 0 cases was reported at Braak stage IV (*p* < 0.05), in line with observations from biochemical measurements. When controlling for age, no significant correlations were observed for AT-8 phospho-tau for either non-AD (Braak stage 0–IV) or AD (Braak stage V–VI) groups (*p* > 0.05), although a modest correlation of Aβ plaque load with Braak NFT stage was observed in non-AD controls (Braak 0–IV; *r* = 0.35, p < 0.05, Fig. 2c.ii).

Furthermore, comparisons between IHC-quantified Aβ and AT-8 phospho-tau also reported a correlation only when the data were analysed as a single cohort (Braak stage 0-VI) combining non-AD controls and AD cases (*r* = 0.68, *p* < 0.0001, Fig. 2d.i), and not when examined as a separate data set of non-AD (Braak stage 0-IV) controls or AD (Braak stage V–VI) cases (*p* > 0.05, for both, Fig. 2dii + iii).

Collectively, the data largely suggests that correlative relationships reported within the overall cohort likely stems from group effects driven by the general increase of pathological hallmarks between non-AD and AD groups and not as an incremental increase in line with progressive Braak stages. Such observations contrast with the findings of the biochemical investigation (see Table 2 for summary).

### Quantification of intracellular Aβ and AT-8 phospho-tau

**Fig. 3:**
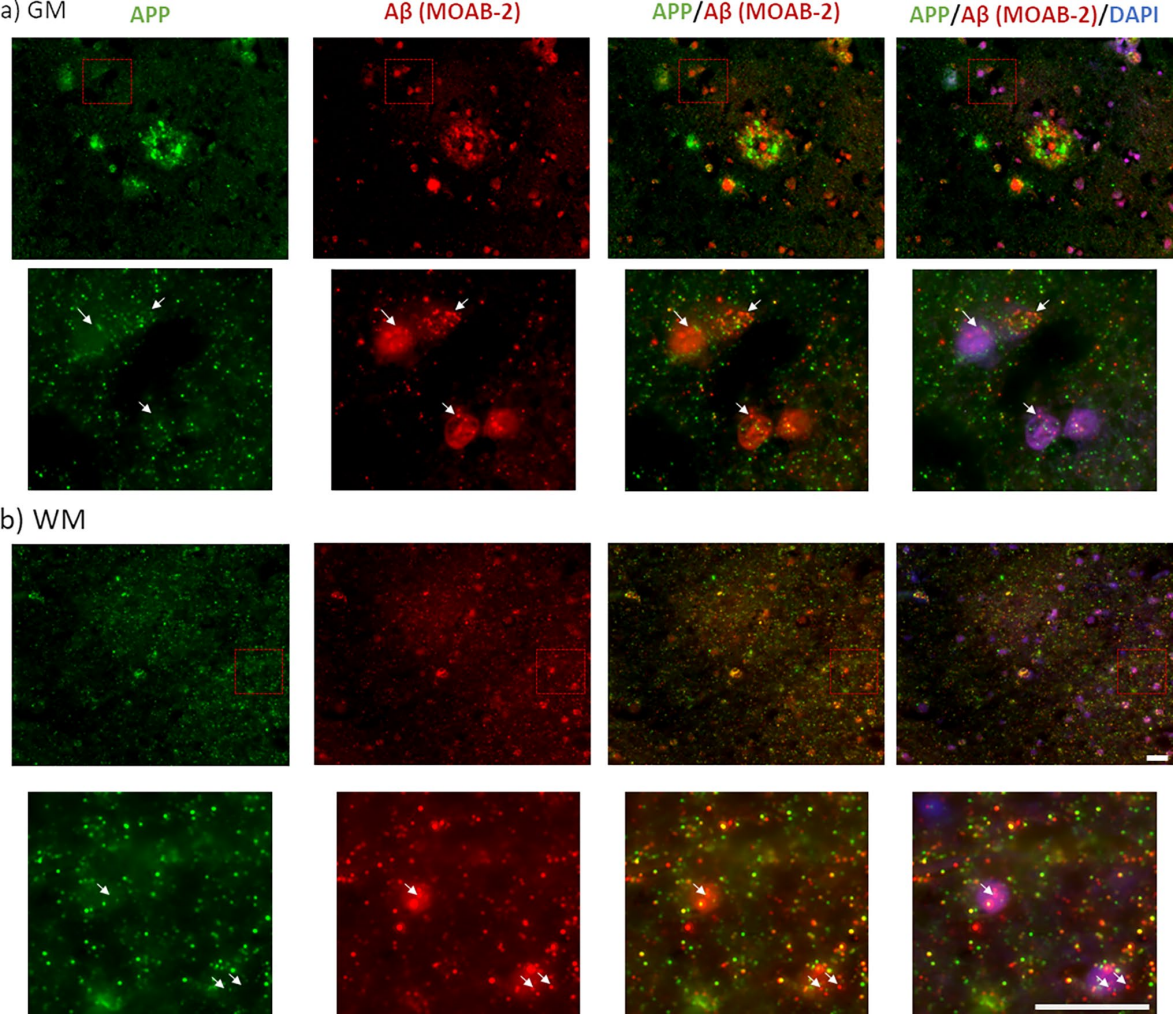
Immunohistochemical distinction between MOAB-2 labelled Aβ and APP immunoreactivity in the frontal cortex of an AD case. Example micrographs of APP (N-terminal-APP antibody) and Aβ (MOAB-2) from an AD case, in the grey (GM; **a**) and white matter (WM; **b**). Note the distinctive labelling of subcellular pools within insert and differential labelling of plaques (in **a**) Scale = 20 µm

The absence of correlations between IHC measures of AT-8 and plaques (Fig. 2), despite a robust correlation with biochemical measures of AT-8 and total Aβ measures from crude tissue lysates (Fig. 1), suggests the possible inclusion of additional non-plaque Aβ sources within the biochemical quantification. The application of MOAB-2 Aβ antibody to fixed post-mortem human brain tissue sections was used, as this labelled both extracellular plaques and intracellular pools of Aβ (Fig. 3). Comparisons of APP and MOAB-2 Aβ labelling demonstrated a clear distinction in subcellular and plaque labelling in both GM (Fig. 3a) and WM (Fig. 3b). This is in line with previous reports demonstrating that MOAB-2 does not bind to APP and other non-Aβ metabolites [33, 74] and is strongly supportive of the specificity of MOAB-2 for the labelling of intracellular Aβ as well as extracellular Aβ.

**Fig. 4:**
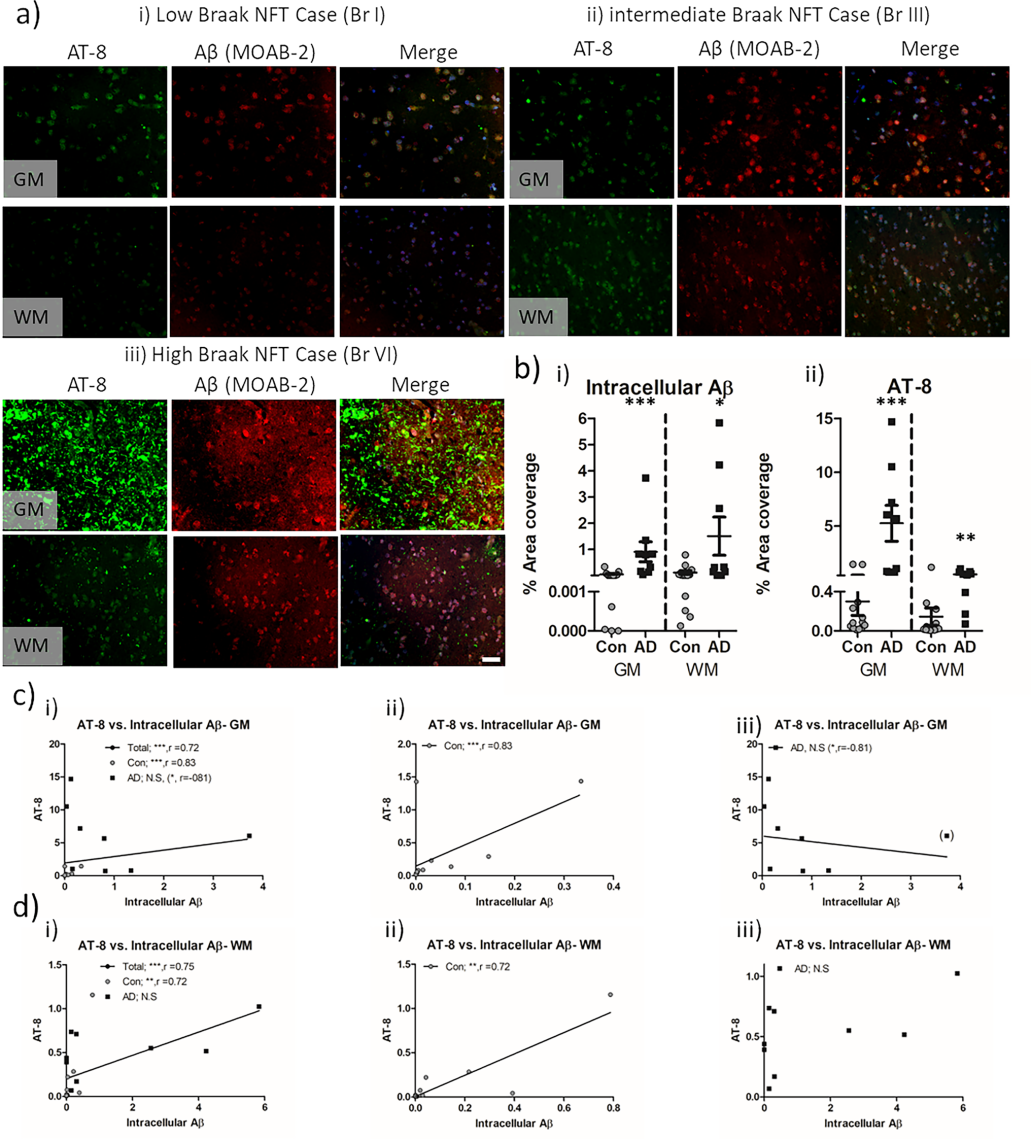
Immunohistochemical quantification of intracellular Aβ and AT-8 tau in the frontal cortex of non-AD and AD cases. **a** Example micrographs of AT-8 phosphorylated tau and MOAB-2 labelled intracellular Aβ in GM and WM of low (*i*), intermediate (*ii*), and high (*iii*) Braak stage cases. **b** Quantification of intracellular Aβ (*i*) and AT-8 phospho-tau (*ii*) expressed as percentage area coverage in control (Con, *n* = 13) and Alzheimer’s disease (AD, *n* = 9) cases. Combined (*i*), Con (*ii*) and AD (*iii*), spearman’s correlations (*r*) between AT-8 and plaques in the GM (**c**) and WM (**d**). Note for the identification of a statistical outlier in **c**, the data point is bracketed and subsequent analysis within correlation excluding the case is reported within brackets. N.S. = not significant, * = *p* < 0.05, ** = *p* < 0.01, *** = *p* < 0.001 and **** = *p* < 0.0001. Scale = 20 µm

Accordingly, a subset of the cohort was stained with MOAB-2, such as to specifically measure intracellular Aβ by means of excluding plaque-based immunoreactivity. Here, a progressive increase in intracellular Aβ staining in line with increase in AT-8 immunoreactivity was observed, both in the GM and WM (Fig. 4a). When intracellular Aβ was quantified according to % area coverage, a significant increase in the levels was observed in AD cases compared to controls (Fig. 4b.i, *p* < 0.001 in GM and *p* < 0.05 in WM). Similarly, AT-8, as measured by immunofluorescence, was again elevated in AD cases (Fig. 4b.ii, *p* < 0.01 in GM and WM). Both AT-8 (*r* = 0.60, *p* < 0.01 in GM and *r* = 0.53, *p* < 0.05 in WM) and intracellular Aβ (*r* = 0.44, *p* < 0.05 in GM) correlated with Braak stage when controlling for age, although WM intracellular Aβ did not correlate with Braak stage. Equally when considered as non-AD controls or AD cases in isolation, no correlation with Braak stages and AT-8 or intracellular Aβ was reported. Regardless, correlative measures between intracellular Aβ and AT-8 phospho-tau reported a significant relationship across the entire cohort (Braak stages 0–VI; in GM, *r* = 0.72 and in WM, *r* = 0.75, *p* < 0.001 for both, Fig. 4c.i + d.i) and in control cases only (Braak stage 0–IV; in GM, *r* = 0.83 and in WM, *r* = 0.72, *p* < 0.01 for both, Fig. 4c.ii + d.ii). When considering only the AD cases (Braak stages V–VI), no significant correlation of intracellular Aβ with AT-8 phospho-tau was observed in either the GM or WM (Fig. 4c.ii + iii). However, notably, in the GM, an inverse trend was observed (*r* = −0.61, *p* = 0.08). Identification and exclusion of a prominent outlier (as per Grubbs test criteria) reported an inverse correlation between intracellular Aβ and AT-8 in the GM of AD cases (*r* = −0.83, *p* < 0.05, Fig. 4c.iii). Additional correlations between GM measures of AT-8 phospho-tau and intracellular Aβ and those in WM were evident at the level of the entire cohort as well as that of controls, but not in AD cases alone (see summary Table 2). Together, the data indicate a change in relationship between phospho-tau and intracellular Aβ in AD cases compared to non-AD controls.

**Fig. 5:**
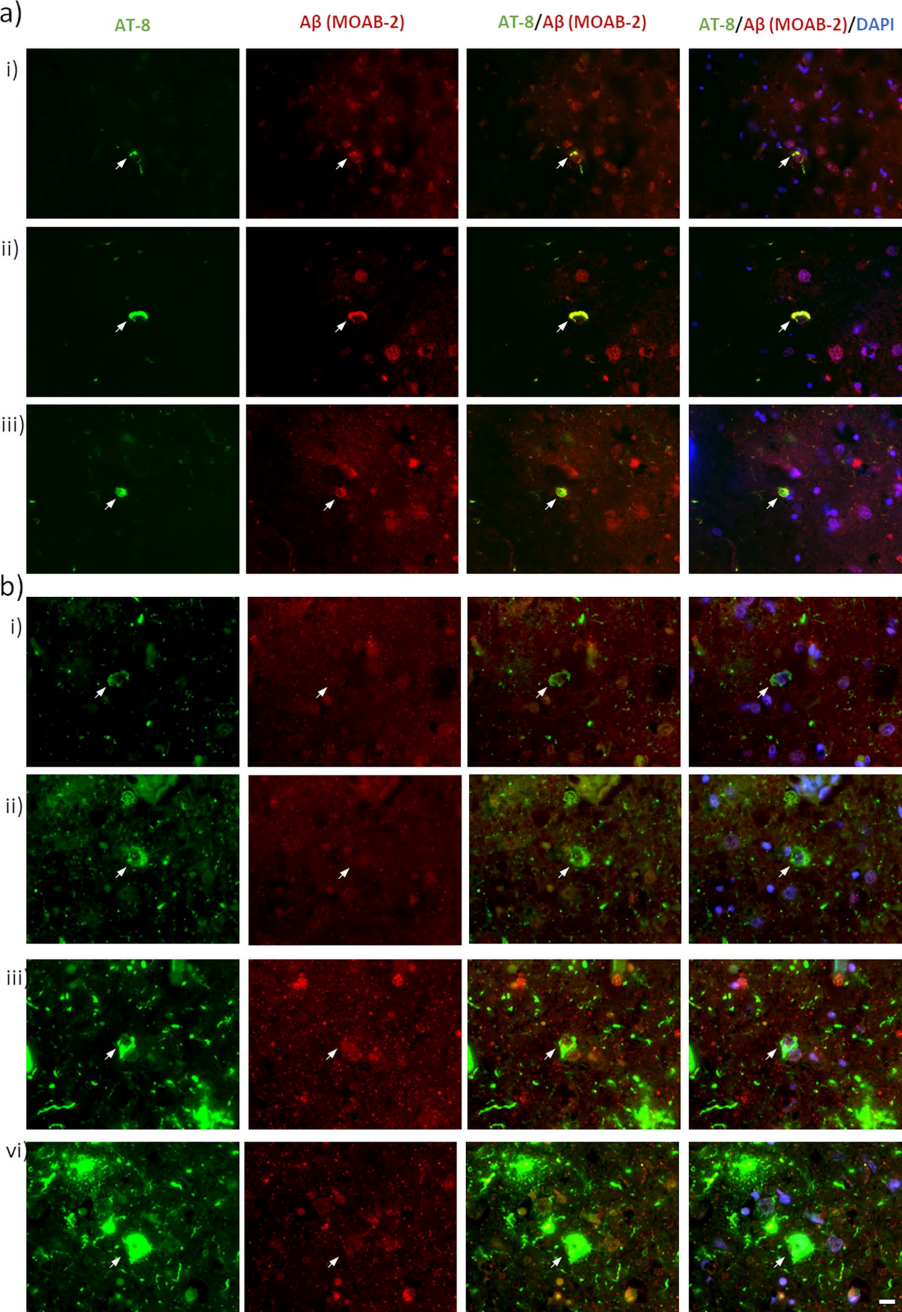
Rare instances of intracellular Aβ and tau colocalization. Micrographs demonstrating the spatial relationship between phospho-tau and intracellular Aβ accumulations. **a** Examples of rarely observed robustly overlapping intracellular AT-8 and Aβ immunoreactivity. Images captured from a non-AD control Braak stage IV (*i* + *ii*) and an AD case Braak stage VI (*iii*). **b** Examples of commonly observed intracellular AT-8 signals in the absence of a notable increased in intracellular Aβ. Images captured from non-AD control Braak stage IV (*i* + *ii*) AD cases Braak stage VI (*iii* + *vi*). Scale = 10 µm

Despite the strong association of intracellular Aβ with AT-8 phospho-tau, spatial colocalization of the two was not common. Robust overlap between phospho-tau and Aβ was only seen in a few rare instances (Fig. 5a). More consistently across the entire cohort, prominent accumulations of AT-8 phospho-tau within neurons occurred in the absence of notable co-localisation of intracellular Aβ (Fig. 5b). Thus, it would appear that AT-8 phospho-tau and intracellular Aβ rarely co-aggregate within the same cell.

## Discussion

This study reports a correlation between biochemical total Aβ and AT-8 phospho-tau measures. Such a relationship was not reproduced when comparing IHC based quantification of extracellular Aβ plaques and AT-8 phospho-tau, yet was observed when considering IHC measures of AT-8 phospho-tau and intracellular Aβ, in non-AD controls. Together, the data suggests a close preclinical/prodromal relationship between non-plaque Aβ and tau in cases of low AD neuropathic change, which is at least partially due to the accumulation of intracellular Aβ and its potential influence on tau phosphorylation. Yet in clinical AD cases, in line with the expansion of pathology, additional factors may diminish the impact of intracellular Aβ on tau pathology.

### Regional correlation of tau and Aβ

A long-standing critique of the amyloid cascade hypothesis has been the disconnect between NFT and plaque burden within a given brain region of either non-AD controls or indeed AD cases [2, 24]. However, many biochemical approaches have previously found correlations between Aβ, either total Aβ or specifically Aβ_1-42_, and a range of phosphorylated and oligomeric tau markers within the GM of a given cortical region [30, 39, 41, 54]. Furthermore, disease-dependent changes in white matter Aβ levels have also been previously observed using Aβ_40_ and Aβ_42_ ELISAs [17] and likewise hyperphosphorylated tau has also been observed biochemically within the white matter of AD cases [21, 73].

Regardless, in the present study, the levels of AT-8 reactive phospho-tau and total Aβ increased in line with Braak stage progression, in both the frontal grey and white matter of non-AD and AD cases. Equally, positive correlations between total Aβ levels and AT-8 were observed in both controls and AD cases. Such findings are consistent with our prior observations in a separate cohort when examining the lateral temporal lobe [39, 41]. Whilst causality cannot be determined, the reported relationships are consistent with many in vitro experiments in which the application of Aβ to various cellular preparations results in downstream tau phosphorylation [20, 34, 77]. Moreover, support for the interaction of Aβ with tau pathology can be gained from studies reporting that interventions targeting Aβ levels consequently reduce tau pathology both in vitro and in vivo models [45, 75] as well as in biofluids obtained from human clinical trials [7, 19, 47].

Conceptually, the generation of tau pathology within a given brain region may be intra-regional as a consequence of local cellular stressors, including elevated Aβ levels or alternatively as a consequence of extra-regional stressors, acting at projecting synaptic terminals, such as distal plaques [29], synaptic Aβ pools [22] and self-propagating tau seeds [21]. Certainly, here the strong correlation of total Aβ and intracellular Aβ with AT-8 signals within non-AD controls would be supportive of the intra-regional generation of pathology. Yet, it remains plausible that extra-regional factors may influence regional pathology. Given that such factors may potentially be detectable from the long-range projections contained within the associated WM samples, the positive correlative measure between WM and GM pathology across the cohort would appear to support this. However, as associations between WM Aβ and GM tau pathology were absent when considered in either non-AD controls or AD cases alone, distal Aβ pools, at least based on the current measures, do not appear to fully account for somatic AT-8 pathology. Similarly, prion-like pathological spread is unlikely to be a prominent underlying factor driving low AD neuropathic change, as an absence of seed competent tau within the frontal cortex of control cases has been previously reported [73]. In clinical AD cases, however, and in line with the progression of global pathology, distal factors may contribute to the pathology in a given region. These additional factors would diminish the relative contribution of regional Aβ to the production of phospho-tau species and as such may in part explain the loss of a correlative relation between in intracellular Aβ and phospho-tau pathology in AD cases.

Whilst all factors driving frontal cortex tau pathology cannot be completely deciphered here, no correlation was observed between plaque Aβ and AT-8 phospho-tau when measured histologically, in either non-AD controls or AD cases. Thus, the data demonstrates the independence of somatic phospho-tau pathology from localized regional plaque load and suggest that biochemical correlations between Aβ and tau are driven by regional non-plaque sources of Aβ. The disconnect between biochemical measures of total Aβ and IHC measures of plaques likely originates from the exclusion of intracellular Aβ pools, as is common practice when assessing Aβ burden as part of a neuropathological assessment [68].

### Intracellular Aβ and tau

Historically, intracellular Aβ has been difficult to quantify, largely due to the cross-reactivity of Aβ antibodies with APP and other intermediate APP metabolites. However, several commercial Aβ antibodies are available, including MOAB-2 which shows no cross-reactivity with APP under many conditions [33, 74]. The production of Aβ via endosomal APP cleavage [36, 59] clearly supports the intracellular generation of Aβ. With age, change in the relative production of Aβ peptide length and altered trafficking mechanisms [35, 42] may act synergically to enhance the retention or reuptake of Aβ, leading to its intracellular accumulation [43]. Accordingly, post-mortem examination of the entorhinal cortex and hippocampus of non-diseased non-AD cases suggests an increase in intracellular Aβ in line with increasing age [23, 71] and furthermore AD animal models also show an age-related accumulation of intracellular Aβ [72, 74].

Here, when selectively measuring intracellular Aβ, a positive correlation between Aβ and AT-8 in IHC measures in the frontal cortex of non-AD controls was observed. However, in the work by others, no such relationship has been observed in the entorhinal cortex [70, 71]. Such discrepancies may relate to the regions of the brain selected for investigation.

The entorhinal cortex is one of the earliest affected cortical regions with tau pathology, but also with the accumulation of intracellular Aβ [35], and thus represents an area of more advanced AD-related pathology relative to that of the frontal cortex. Accordingly, in vitro cellular and in vivo mouse model studies of induced Aβ pathology have observed a redistribution of somatic intracellular Aβ into distal processes over time [61, 64]. Such a translocation of intracellular Aβ pools may mean that an accurate post-mortem determination of total intracellular Aβ, may not be captured within a given field of interest focused on neuronal cell bodies, specifically in early affected regions. Nevertheless, within the prefrontal cortex, a region which does not demonstrate robust age-related NFT tau pathology and is not burdened with NFTs until late into the Braak NFT staging criteria (Braak V–VI), the modest pre-tangle tau pathology generated in this region may be largely dependent on an intracellular Aβ-mediated mechanism within non-AD controls.

Such a mechanism may become modified under pathological conditions within AD cases, as a consequence of extra-regional influences (as discussed above) or indeed by the intracellular distribution of Aβ away from the soma into projections. Interestingly in GM, an inverse correlation between intracellular Aβ and phospho-tau was apparent in AD cases, although ultimately not significant. This observation is consistent with the reported decline in intracellular Aβ levels alongside increased Aβ plaque deposition in mice models [57] as well as in cross-sectional observations in Down’s syndrome brains [53] and cases of late-stage NFT-mediated neurodegeneration [70]. Though speculative, it is plausible that elevation of intracellular Aβ precedes, and indeed acts as a source for, extracellular plaque deposition, with the excessive deposition of plaques at later stages subsequently reducing intracellular Aβ levels as observed in animal models [57]. In turn, tau pathology may continue to grow due to the influence of self-propagation via seed component tau species or indeed the influence of distally located intra/extracellular pools of Aβ (as discussed above).

Given emerging evidence from clinical Aβ antibody trials [61], which support the targeting of soluble fibrillar Aβ species to consequently reduce tau pathology, further understanding the degree of interaction between Aβ and tau will provide greater insight into the mechanisms of AD-related pathogenesis. Equally, in light of the facilitation of fibril seeding by the existence of pre-existing tau phosphorylation/pathology in mice [16, 25, 32], the targeting of pre-tangle soluble tau elevations in late-stage-affected brain regions, may protect against tau seed infiltration as part of AD disease progression and may provide an effective stalling of the condition.

### Limitations

Despite these observations, the current study, and its findings, must be taken within the context of its limitations. As with most post-mortem studies, assumptions have been made to enable the extrapolation of cross-sectional analysis to longitudinal disease progression. Specifically, correlations between experimental measures and Braak NFT staging have assumed a linear progression of pathology with each stage and that regional pathology is systematically modified in relation to global pathology. Furthermore, it is assumed that such factors are consistent across individuals. Moreover, group-specific correlative analysis conducted between experimental measures and Braak stages of 0–IV for controls and V–VI for AD may be relatively weak in reliability, particularly for AD cases in which correlations are made only between two points. Nevertheless, such analysis provides a granular insight into the change of tau and amyloid markers in relation to disease progression, whilst the strongest and most pertinent analysis is taken from correlative analysis of AT-8 phospho-tau and amyloid measures across individuals. Finally, here correlative relationships of Aβ and tau pathology are based on the AT-8 phospho-tau antibody. Whilst AT-8 is the gold standard antibody for neuropathological assessment of tau pathology, it is possible that phosphorylation at additional or alternative residues may be more directly associated with the influence of Aβ on tau pathology. Nevertheless, our conclusions are not intended to suggest that regional intracellular Aβ is the only factor in driving tau pathology, rather that intracellular Aβ influences AT-8 phospho-tau pathology on a regional basis, particularly at the early stages of pathological development. Such a role for intracellular Aβ may in part explain the reported biochemical correlations between tau pathology and Aβ, even in the absence of a correlation between AT-8 tau pathology and Aβ plaque load, as per IHC.

## Conclusions

Collectively, this study demonstrates the robust correlation of AT-8 reactive tau and Aβ in the frontal cortex of both non-AD controls and AD cases when measured biochemically. Given that such linear increases in Aβ plaques and AT-8 pathology are not observed when quantified via IHC, the study demonstrates the potential influence of non-plaque Aβ in the intra-regional generation of tau pathology in non-AD control cases. Specifically, the occurrence and accumulation of intracellular Aβ may contribute to the production of tau pathology, in cases of low AD neuropathic change. This finding is supportive of the amyloid cascade hypothesis, yet in late-stage AD cases such a relationship may be diminished, with additional factors contributing to tau pathology, at least within the frontal cortex. Critically, the observation of a localized relationship between Aβ and phospho-tau in cases with low Braak NFT stages implies that there is a degree of regionally generated AD-related pathology, which may be tolerated within a physiological range. Following the age-related accumulation of pathology, this regionally produced burden may prime the region for the influence of extra-regional factors such as distal intra- and extracellular pools of Aβ as well as the invasion of seed competent forms of tau, which have originated from connected regions.

### Supplementary Information

Below is the link to the electronic supplementary material.

Supplementary file1 (DOCX 22 KB)

## Data Availability

All data sets are available from the corresponding author upon request.

## Abbreviations

Aβ: Amyloid beta
APP: Amyloid precursor protein
AD: Alzheimer’s disease
BA: Brodmann area
CERAD: Consortium to Establish a Registry for Alzheimer’s Disease
Con: Controls
DAB: 3,3′-Diaminobenzidine
DPX: Dibutylphthalate polystyrene xylene
FAD: Familial Alzheimer’s disease
GM: Grey matter
HRP: Horse radish peroxidases
IHC: Immunohistochemical
LB: Lewy body
NIA-AA: National Institute of Ageing-Alzheimer’s Association
NBTR: Newcastle Brain Tissue Resource
NFTs: Neurofibrillary tangles
PART: Primary age-related tauopathies
PET: Positron emission tomography
TBS: Tris-buffered saline
TBST: Tris-buffered saline with Tween-20
TMA: Tissue micro-array
WM: White matter
